# Evaluating the Relationship between Circadian Rhythms and Sleep, Metabolic and Cardiovascular Disorders: Current Clinical Evidence in Human Studies

**DOI:** 10.3390/metabo13030370

**Published:** 2023-03-01

**Authors:** Maria Mentzelou, Sousana K. Papadopoulou, Dimitrios Papandreou, Maria Spanoudaki, Antonios Dakanalis, Georgios K. Vasios, Gavriela Voulgaridou, Eleni Pavlidou, Maria Mantzorou, Constantinos Giaginis

**Affiliations:** 1 Department of Food Science and Nutrition, School of Environment, University of Aegean, 81400 Myrina, Lemnos, Greece; 2 Department of Nutritional Sciences and Dietetics, School of Health Sciences, International Hellenic University, 57400 Thessaloniki, Greece; 3 Department of Health Sciences, College of Natural and Health Sciences, Zayed University, Abu Dhabi P.O. Box 144534, United Arab Emirates; 4 Clinical Dietetics & Nutrition Department of 424 General Military Hospital, New Efkarpia Ring Road, 56429 Thessaloniki, Greece; 5 Department of Medicine and Surgery, University of Milano Bicocca, Via Cadore 38, 20900 Monza, Italy

**Keywords:** circadian rhythms, metabolic disorders, cardiovascular diseases, obesity, diabetes mellitus, sleep quality

## Abstract

Circadian rhythms are generated by the circadian clock, a self-sustained internal timing system that exhibits 24-h rhythms in the body. Many metabolic, cellular, behavioral and physiological processes are regulated by the circadian clock in coordination with environmental cues. The present study is a comprehensive review of the currently existing evidence concerning the relationship between circadian rhythms and sleep, metabolic, and cardiovascular disorders. We thoroughly searched the online databases PubMed, Scopus, and Web of Science to find the existing clinical studies from the last twenty-three years (2000–2023). Circadian misalignment was found to be associated with an increase in the risk of metabolic disorders, cardiovascular diseases, and obesity, as well as inadequate sleep quality. In this review article, all the included studies had a strength protocol design and all of them were conducted on humans. However, the most common limitations of them were the small sample size and the short time of the intervention. In conclusion, managing the factors that disrupt the optimal function of central and peripheral clocks can help to reduce the risk of metabolic and cardiovascular diseases, improving also sleep quality. Future studies should further explore the underlying mechanisms of the interconnections between circadian clocks and sleep, metabolic, and cardiovascular disorders. This may provide new opportunities for advance chronotherapy approach.

## 1. Introduction

The circadian system organizes metabolism, physiology, and behavior in a daily cycle of circadian rhythms. Circadian (Latin “circa” meaning around and “diem” meaning a day) clock orchestrates biological processes in adjusting to daily environmental changes [1,2,3]. The circadian system comprises a central pacemaker in the brain and a series of clocks in peripheral tissues throughout the body, including liver, muscle, and adipose tissue. The integrating circadian systems with the light/dark environment involves a widely distributed network of local tissue clocks within both brain and periphery [1,2,3]. Circadian rhythms are modulated by endogenous (genetic, physiological) as well as environmental (light) and behavioral (activity, feeding) factors, food availability, glucocorticoid level, and temperature [1,2,3]. 

The main function of the circadian clock Is to entrain the organism with the environment (external synchronization) and maintain endogenous physiological process temporally organized (internal organization). Various body functions have a period of activity where these functions are at their peak and a period of inactivity when the functions are nadir [4,5,6].

Any forced disruption to the normal sleep patterns, e.g., shift work or frequent traveling over two or more time zones (jetlag), can lead to circadian misalignment and have been associated with various metabolic diseases [7,8,9,10]. The chronic metabolic disorders are a major public health concern worldwide. In this aspect, sleep deficiencies and circadian disruption related with metabolic dysregulation can contribute to weight gain, obesity, and type 2 diabetes potentially by modifying timing and quantity of food intake, diminishing energy balance, inflammation, and impairing glucose tolerance, and insulin sensitivity [8,9]. In a cross-sectional study in 2012, sleep loss and circadian rhythm abnormalities were considered as underlying causes of adverse metabolic health, such as diabetes type 2 [7]. In a recent meta-analysis of 38 observational studies conducted on 128,416 individuals, rotating shift workers had stronger odds of metabolic syndrome (MetS) compared to other shift workers, supporting an association between shift work and MetS and different effects for sleep, gender, and type of shift work [10].

Circadian rhythms have an important role in the regulation of cardiovascular physiology and health. Peripheral clocks are present in each of the cardiovascular cell types, regulating a lot of aspects of functions, blood pressure, heart rate, circulating catecholamines, blood coagulation markers, and vascular endothelial function [11,12]. As it concerns the light at night, it has been shown to reduce the secretion of nocturnal pineal melatonin and disrupt the circadian rhythms. In this aspect, a substantial prospective study in 58,692 Chinese elders showed that higher levels of night-time light intensity were positively associated with measures of carotid atherosclerosis [13]. Moreover, the risk of coronary heart disease (CHD) may be increased by the exposure to night-time light due to the disruption of circadian rhythms, which affect endothelial function, thrombus formation and triggering inflammatory responses [11,13].

In the general population, observational studies have also found that short sleep and sleep deprivation increased appetite and obesity [14]. Moreover, adipose tissue is a critical modulator of metabolic and cardiovascular health [15,16]. For instance, 24-h oscillation in levels of glucose and fAs may in turn influence expression of circadian genes and rhythmic transcriptional outputs involved in glucose homeostasis, food intake, and energy expenditure [17,18].

Sleep timing is known to shift later during puberty, with the most delayed timing around age 20, and then slowly shifts earlier over subsequent decades. Variation in sleep behavior can complicate identification of endogenous chronotype due to the influence of genetics. Circadian disorders arise when sleep–wake patterns, influenced by the circadian clock, are not in sync with desired sleep time, leading to a sleep or wake complaint [19,20,21]. The term ‘circadian rhythm sleep disorder’ refers to a chronic condition in which an individual’s circadian rhythm of sleep and wakefulness is out of phase with the conventional environmental patterns [22]. These include intrinsic circadian disorders, such as advanced sleep–wake phase disorder, delayed sleep–wake phase disorder, non-24 sleep–wake rhythm disorder, and irregular sleep–wake rhythm disorder; and extrinsic circadian disorders, such as shift work and jet lag disorder [23]. The most common symptom of them is that the patient has not had the quality of sleep that is desired, needed, or expected. The internal biological clock is inappropriately aligned with sleep, and this justifies the fact that wake episodes can occur at undesired times [22].

The above interconnections between the circadian system, metabolism, and behavior point to the importance of chronobiology for preventing and treating diabetes, obesity, and hyperlipidemia. A better understanding of the circadian system disorder is required for the development of strategies to prevent and decrease the risk of health issues [19,24,25,26,27,28,29]. In view of the above considerations, this review critically examines the existing literature on the relationships between circadian rhythms and sleep, metabolic, and cardiovascular disorders, highlights the limitations of the current body of research, and provides suggestions for future research.

## 2. Methods

This is an in-depth, comprehensive review of the currently existing evidence concerning the interrelationship between circadian rhythms and sleep, metabolic, and cardiovascular disorders. In fact, the most accurate scientific databases, e.g., PubMed, Scopus and Web of Science were comprehensively searched utilizing relative words, such as “circadian rhythm”, “metabolic disorders”, “diabetes mellitus”, “environmental factors”, “dietary habits”, “circadian disruption”, “cardiovascular diseases”, “sleep quality”, “sleep duration” to find the already existing clinical studies from the last twenty-three years (2000–2023). The results were filtered according to relevance and the most relevant ones were selected and quoted below. Only clinical human studies with a strength protocol design and written in English language were included in this review. Review articles, case reports and abstracts in congress proceedings were excluded from the final analysis.

## 3. Results and Discussion

### 3.1. Circadian Rhythms and Metabolic Diseases

There are a lot of clinical studies that assessed the effect of circadian disruption in human metabolism. The currently existing studies are summarized in Table 1.

Leproult et al. [30] examined in 26 adults whether circadian misalignment may have adverse cardiometabolic effects independently of sleep loss. The interventions involved 3 days with 10-h bedtimes, followed by 8 days with 5-h bedtimes, with bedtimes always centered at 0300 h (0030–0530 h, circadian alignment) or with bedtimes delayed by 8.5 h on 4 days (0900–1400 h, circadian misalignment) [30]. This study assessed sleep times, circadian phases, food intake and cardiometabolic variables. When sleep was restricted to 5 h, participants sleeping mostly during the day (circadian misalignment) had 47% greater reduction in insulin sensitivity compared with 34% reduction in insulin sensitivity of participants sleeping during the night [30]. This study also documented an increase in high-sensitivity C-reactive protein (hs-CRP) after sleep restriction in the misaligned groups, which was higher compared to the aligned groups. The increased risk of diabetes and cardiovascular disease is unlikely to be solely due to sleep loss and would not be fully mitigated by strategies designed to preserve sleep duration [30]. The findings of this clinical trial are unequivocal due to the carefully execution of the protocol; however, the sample size is quite small [30].

In another study, Morris et al. evaluated the separate effects of the behavioral cycle, circadian phase, and circadian misalignment on glucose metabolism [31]. One protocol included circadian misalignment and the other maintained circadian alignment in 14 healthy adults. In the circadian alignment protocol, the participant’s sleep opportunity occurred between 11:00 and 19:00 h for days 1–8. In the circadian misalignment protocol, the participant’s sleep opportunity occurred between 11:00 and 19:00 h for days 1–3. On day 4 of the circadian misalignment protocol, the participant’s behavioral cycles were shifted by 12 h, and this was remained until the end of that protocol (day 8) [31]. The results of this study supported evidence that separate effects of the endogenous circadian system and of circadian misalignment, independent from effects of the behavioral cycle, may impact on glucose tolerance in humans [31]. These variations in glucose tolerance may be ascribed, at least in part, to different mechanisms: during the biological evening by decreased pancreatic β-cell function (27% lower early-phase insulin) and during circadian misalignment presumably by reduced insulin sensitivity (elevated postprandial glucose despite 14% higher late-phase insulin) without alterations in early-phase insulin [31]. The fact that the behavioral and environmental conditions remain the same is an undeniable strength, but this study assessed only two behavioral and circadian cycle phases without determining the behavioral cycle on glucose metabolism. However, this study may help the development of behavioral and circadian strategies that could improve glycemic control in day-active people and night workers [31].

To determine whether these findings persist in the shift-work population, the impact of circadian misalignment in a real-life shift worker was evaluated, as well. The intervention included simulated night work comprised of 12-h inverted behavioral and environmental cycles (circadian misalignment) or simulated day work (circadian alignment) [31,32]. For this reason, the above studies measured blood pressure and inflammatory markers across the 24-h behavioral and light/dark cycles when the behavioral and environmental cycle was aligned and misaligned with the endogenous circadian system [31,32]. Their results showed that short-term circadian misalignment increased 24-h blood pressure [31,32]. Moreover, circadian misalignment reduced sleep blood pressure dipping during sleep opportunities, which may be also an independent predictor of adverse cardiovascular events and all-cause mortality [31,32]. Moreover, circadian misalignment increased the inflammatory markers CRP, tumor necrosis factor (TNF)-α, resistin, and interleukin (IL)-6. This suggests that the internal circadian time of food intake may be an important factor to consider in shift workers [31,32]. The strength of these studies included the highly controlled laboratory protocol, which was able to determine the independent impact of circadian misalignment, while the sample size was small [31,32].

Furthermore, another study tested the impact of circadian misalignment, similar to that experienced by real-life shift workers, on 24-h levels of hs-CRP and blood pressure, which are risk factors for cardiovascular disease [33]. For this reason, this study measured hs-CRP and blood pressure levels across the 24-h behavioral and light/dark cycles when the behavioral and environmental cycle was aligned and misaligned. This study also found that short-term circadian misalignment, increased 24-h hs-CRP and 24-h blood pressure in chronic shift workers, which both may increase the prevalence of cardiovascular disease [33].

Moreover, Qian et al. [34] assessed the separated effects of circadian misalignment from effects of circadian phase and environmental/behavioral factors on glucose control in 14 healthy adults using a randomized, cross-over design with two 8-day laboratory protocols [34]. Both protocols involved 3 baseline inpatient days with habitual sleep/wake cycles, followed by 4 inpatient days with the same nocturnal bedtime (circadian alignment) or with 12-h inverted behavioral/environmental cycles (circadian misalignment) [34]. These findings showed that the endogenous circadian system and circadian misalignment, after controlling for behavioral cycle influences, exerted independent and differential impacts on insulin sensitivity and pancreatic β-cell function in diurnally active people as well as night shift workers [34]. The circadian system mainly decreased both dynamic and static β-cell responsivity, while circadian disruption mainly reduced insulin sensitivity without impairing pancreatic β-cell function [34]. The design of this study allowed to distinguish the function of β-cell due to circadian phase and/or circadian disruption. However, the highly controlled diet throughout the study decreased the potential effect of variations in energy intake [34].

Furthermore, Wefer et al. [35] assessed insulin sensitivity by the hyperinsulinemic and euglycemic clamp. Each participant underwent a 3-day control protocol of circadian alignment and after a 3.5-day protocol of circadian misalignment [35]. The findings of this study pointed that short-term circadian misalignment resulted in a significant decrease in insulin sensitivity that was mainly ascribed to the impairment in insulin-stimulated nonoxidative glucose disposal [35]. In addition, the molecular biological clock in skeletal muscle was misaligned relative to the behavioral routine, suggesting that skeletal muscle may be not adjust to a new day–night rhythm within 3 days [35]. Circadian misalignment also led to higher fasting free fatty acids (FFAs) levels, fasting plasma glucose levels, and lower triglyceride levels [35]. Limitations of this study were that it only included male volunteers and the lack of information on sleep quality, which can decrease insulin sensitivity. Future studies should examine whether similar results can be found in individuals at risk for diabetes mellitus [35].

Madjd et al. [36] evaluated the effects of higher energy intake at lunch compared to dinner on weight loss and also on indexes of carbohydrate and lipid metabolism in overweight and obese women (N = 80) who were attending a weight-loss program for 12 weeks [36]. The findings of this study showed that consumption of the main meal at lunch led to more weight loss and a greater improvement in insulin sensitivity as measured by homeostasis model assessment-estimated insulin resistance (HOMA-IR) and fasting insulin concentrations than did eating the main meal at dinner in overweight and obese women [36]. Notably, the findings of this study may have practical implications, indicating that the consumption of a main meal at lunch and not at dinner could improve weight loss when people use a weight-loss program [36]. The principal strength of this study was at first, that it was a randomized, outpatient, clinical trial, in which subjects were following a comprehensive diet plan for weight control [36]. Secondly, the provision of a free diet plan and a daily telephone call from a dietitian to each subject was an encouragement in both groups. In contrast, the main limitation of this study was the short-term intervention period [36].

Bandín et al. [37] analyzed the differences between taking an early (13.00) and late (16.30) lunch in 32 women. In each protocol, participants were provided with standardized meals (breakfast, lunch, and dinner) during the two meal intervention weeks and were studied under two lunch-eating conditions: Early Eating (EE; lunch at 13:00) and Late Eating (LE; lunch 16:30) [37]. The results of this study showed that delaying the timing of an identical meal for a week resulted in decreased resting energy expenditure prior to the meal, unchanged postprandial energy expenditure, reduced fasting carbohydrate oxidation, lowered glucose tolerance, blunted daily profile of free cortisol concentrations, and slowed down thermal effect of food [37]. Chronically, eating at a later time of day may create metabolic disturbances of a larger magnitude and could be implicated in the metabolic alterations that characterize late eaters [37]. However, further studies are strongly required to measure the effect of early and late eating on energy expenditure across 24 h to assess whether meal timing may affect energy expenditure at other times of day. Moreover, there is need to determine the underlying mechanisms, and to resolve whether the endogenous circadian rhythm of cortisol is influenced by meal timing [37].

In the randomized controlled clinical trial of Barger et al. [38], it was assessed whether sleep duration, sleep apnea, and shift work were independent risk factors of cardiovascular diseases. It was found that patients with shorter duration of sleep (less than six hours) had an increased risk of serious cardiovascular events, than those having longer sleep. Patients with obstructive sleep apnea had 12% more risk of cardiovascular events than patients with normal sleep [38]. Overnight shift work had, also, a higher risk than those working in the day. The study design was limited to a self-reported questionnaire, and there was a lack of objective confirmation of these reported sleep-related factors [38]. More to the point, the Berlin questionnaire has not been validated yet in a population of post-acute coronary syndrome patients, but it has been validated in small studies of patients with cardiovascular or cerebrovascular disease [38]. Further research should determine the potential physiological link between sleep duration, sleep disruption, sleep disorders, and cardiovascular risk [38].

Sharma et al. [39] examined the potential effect of rotational shift-work on glucose metabolism Participants underwent an isotope-labeled mixed meal test during a simulated day shift and a simulated night shift, enabling simultaneous measurement of glucose flux and pancreatic β-cell function using the oral minimal model [39]. This study revealed that postprandial glucose concentrations were higher during the night shift; further, the timing of peak insulin and both C-peptide and nadir glucagon suppression after a meal were also delayed due to β-cells weaker responsivity to glucose [39]. In addition, these changes may represent circadian variations in insulin secretory capacity driven by changes in β-cell clock gene expression [39]. The experimental protocol designed very carefully in terms of sleep quantity, energy intake and meal composition [39]. However, further studies are necessary to determine the mechanism(s) of diurnal decline in β-cell function and whether exposure to more prolonged patterns of chronic shiftwork promotes sustained or greater decreases in β-cell function [39].

Jarrin et al. [40] evaluated, also, the potential role of sleep abnormalities and shift work on the development of heart failure due to loss of parasympathetic control. The sample of this study was divided into two groups, with sleep duration shorter <6 h or equal/longer >6 h [40]. Electrocardiogram data derived from polysomnography were applied for obtaining heart rate and heart rate variability during stage 2 and rapid eye movement sleep [40]. The findings of this study showed that patients with short sleep duration insomnia had reduced parasympathetic activity as compared to those with normal sleep duration insomnia [40]. It was also found an increased imbalance between sympathetic and parasympathetic balance [40]. Thus, treating insomnia may reduce the risk of cardiovascular diseases. In the analysis of this study, one strength was the relatively large sample comprised of patients suffering from insomnia on average for 15 years [40]. In contrast, the main limitation was the cross-sectional nature of the study, which precluded the investigation of a causal relation to be assessed between cardiovascular autonomic function, insomnia, and short sleep duration. Moreover, there was no group with normal conditions [40].

### 3.2. Interrelationships between Circadian Rhythms, Sleep Disorder, and Metabolic Diseases

There are a lot of clinical studies that assessed the interrelationship between circadian rhythms, sleep disorder, and metabolic diseases in humans. The currently existing studies are summarized in Table 2.

Wong et al. [41] investigated whether chronotype and social jet lag covaried with components of cardiometabolic risk in a nonpatient sample of midlife community volunteers and whether any such associations persisted after adjustment for correlated variation in health practices, including behavioral and subjective measures of other sleep characteristics. Their results showed that a mismatch in sleep timing between workdays and free days linked to greater cardiometabolic risk, specifically with components of glycemic control, serum lipids, and adiposity. These effects persisted after adjusting for correlated variation in other sleep parameters and with further adjustment for participant health behaviors. Due to the cross-sectional study design, future prospective studies are strongly recommended to extend the present findings [41].

The cross-sectional study of Ritonja et al. [42] was conducted to determine the association between night work parameters (current night work status, night work duration, and night work intensity) and cardiometabolic risk factors and how it differed by chronotype. This study supported evidence that various night work parameters were related to poorer overall cardiometabolic health, including higher waist circumference and BMI, fasting blood glucose, blood pressure, cardiometabolic risk score, and lower low-density lipoprotein (LDL) cholesterol. One strength of this study was the inclusion of both night work intensity and years of shift work duration in the assessment of night work exposures. The use of objective measures of cardiometabolic indices and chronotype also avoided information bias. Limitations included a small sample size and the cross-sectional design of the study. However, further research is needed to make clear the exact biological pathways between rotating night work and cardiometabolic risk [42].

Hulsegge et al. [43], in their cross-sectional study, investigated relations between shift work and various cardiometabolic risk factors and explored these potential relations in different chronotypes. The findings of this study showed that shift work was not related with an increased risk of cardiometabolic risk factors, except for overweight/BMI. Shift work was not associated with an increased risk of cardiometabolic risk factors, except for overweight/BMI. A strength of the study was the wide variety of objectively measured cardiometabolic risk factors. However, more research is needed on the moderating effects of chronotype to establish whether tailored preventive measures by chronotype may be useful for shift workers [43].

Ji Hee Yu et al. [44] examined whether late chronotype could be associated with metabolic abnormalities and body composition in middle-aged Korean men and women independent of sleep profile and lifestyle factors. Evening chronotype was associated with lower lean mass in men and higher fat mass in women. Moreover, evening type was likely associated with a worse metabolic profile than other chronotypes for several reasons. Thus, chronic circadian misalignment could be one of the reasons explaining metabolic derangements in evening types. The main limitation of this study was its cross-sectional design [44].

The meta-analysis of Rui Zhang et al. [45] explored the association between evening chronotype and circadian misalignment with obesity, type 2 diabetes mellitus (T2 DM), and metabolic syndrome (MetS) in non-shift workers. They found that evening chronotype was associated with unfavorable metabolic indicators including higher BMI, higher fasting glucose level, higher total cholesterol level and lower high-density lipoprotein (HDL)-c level compared with morning chronotype. Moreover, higher social jetlag was associated with larger waist circumference compared with smaller social jetlag. Due to the exposure to artificial light and work demand in modern lifestyle, circadian misalignment may be considered as a quite common phenomenon. This study had the strength that it was the first meta-analysis to assess the association between evening chronotype and circadian misalignment and parameters of MetS in non-shift working adults. The limitations of this study were the heterogeneity and the cross-sectional data [45].

Another cross-sectional clinical study examined the circadian integration of glycemic control in a clinical setting to assess the relationship between morningness–eveningness and glycemic control. This study supported evidence that the sleep–wake pattern of the circadian rhythm correlated with inadequate glycemic control along with low health-related quality of life in patients with type 2 diabetes. The main limitations of this study were the lack of classification (morning, evening, neither group) and the small number of participants. Further studies are required to confirm the relationship among sleep–wake patterns, glycemic control, and lifestyle factors, such as dietary habit, physical activity, and smoking habit [46].

Koopman et al. [47] investigated the association of social jetlag with the MetS and T2 DM in a population-based cohort. They observed an association between social jetlag and the metabolic syndrome only for participants with >2 h social jetlag, compared with participants with <1 h social jetlag. In addition, they suggested that the association between social jetlag and MetS was driven by higher glucose and waist circumference. The limitations of this study were the cross-sectional data as well as the incomplete follow-up data [47].

**Table 2:** Clinical studies analyzing the interrelationship between circadian rhythm, sleep disorder, and metabolic diseases.

Study Type	Study Population	Basic Results	References
Clinical Trial	N = 447 adults	Social jet lag is related to a lower HDL-cholesterol level, higher triglycerides, higher fasting plasma insulin, insulin resistance, and adiposity.	Wong et al. [41]
Clinical Trial	N = 325 female adults	Night work, cumulative night work, and night work intensity are associated with a number of cardiometabolic indices, including higher waist circumference, body mass index (BMI), fasting glucose, blood pressure, and cardiometabolic risk score.	Ritonja et al. [42]
Cross-sectional Study	Ν = 1334 adults	Shift work was not significantly related with cardiometabolic risk factors, except for overweight/body mass index	Hulsegge et al. [43]
Cross-sectional Study	N = 1620 adults	Evening chronotype was independently associated with diabetes, metabolic syndrome, and sarcopenia.	Ji Hee Yu et al. [44]
Cross-sectional Study	N = 101 male adults	‘Eveningness’ type male Japanese workers with T2 DM suffer inadequate glycemic control.	Iwasaki et al. [46]
Cross-sectional Study	N = 1585 adults	Social jetlag of ≥2 h was associated with increased risk of MetS and diabetes/prediabetes after adjustment for sex, employment status, and education level	Koopman et al. [47]
Meta-analysis	N = 27 studies	Evening chronotype and social jetlag were associated with obesity and unfavorable metabolic parameters of glucose and lipid metabolism	Rui Zhang et al. [45]

### 3.3. Circadian Rhythms and Cardiovascular Diseases

There are a lot of clinical studies that assessed the relationship between circadian rhythms and cardiovascular diseases (CVDs) in humans. The currently existing studies are summarized in Table 3. As we analyzed before, the randomized controlled trials highlighted the crucial effect in cardiovascular disease, in various aspects [31,32,33,41].

Estarlich et al. [48] investigated whether a circadian pattern in the occurrence of acute coronary syndrome existed and what factors influenced the severity of acute myocardial infraction, its location, the length of hospital stay in patients in Spain, and whether individual risk factors resulted in differing patterns. Acute coronary syndrome seemed to occur more often in the morning hours. Morning was also associated with an increased risk of anterior infarction, which was related to the severity of the disease. The main limitation of this clinical study was the small size of the sample [48].

**Table 3:** Clinical studies analyzing the relationship between circadian rhythms and cardiovascular diseases.

Study Type	Study Population	Basic Results	References
Clinical Trial	N = 14 adults	Glucose tolerance was 17% lower in the biological evening than in the biological morning on test day 1. Circadian misalignment itself increased postprandial glucose by 6%.	Morris et al. [31]
Clinical Trial	N = 9 adults	Circadian misalignment increased 24-h SBP and DBP by 3.0 mmHg and 1.5 mmHg, respectively. Circadian misalignment decreased wake cardiac vagal modulation by 8–15%, as determined by heart rate variability analysis, and decreased 24-h urinary epinephrine excretion rate by 7%, without a significant effect on 24-h urinary norepinephrine excretion rate. Circadian misalignment increased 24-h serum IL-6, CRP, resistin, and TNF-α levels by 3–29%.	Morris et al. [32]
Clinical Trial	N = 447 adults	Social jet lag was related to a lower HDL-cholesterol level, higher triglycerides, higher fasting plasma insulin, insulin resistance, and adiposity.	Wong et al. [41]
Clinical Trial	N = 26 adults	Sleep restriction and circadian misalignment both enhanced cardiovascular risk.	Grimaldi et al. [49]
Retrospective cross-sectional Study	N = 244 adults	It was a circadian pattern of acute myocardial infraction.	Estarlich et al. [48]
Prospective Study	N = 58,692 adults	Outdoor light at night was associated with a higher risk of CHD hospitalizations and deaths.	Sun et al. [13]
Prospective study	N = 189,158 women	The association between duration of shift work and CHD was stronger in the first half of follow-up than in the second half.	Vetter et al. [50]
Prospective study	N = 1992 adults	Individuals with the most irregular sleep duration or timing had >2-fold risk of developing CVD over a median follow-up of 4.9 years.	Huang et al. [51]
Randomized Controlled Trial	N = 9 adults	Circadian misalignment per se increases hs-CRP and blood pressure in shift workers.	Morris et al. [33]

Sun et al. [13] estimated the association between residential outdoor light at night and risk of CHD among older adults in Hong Kong. They found that individuals living in areas of the region with higher levels of outdoor light at night may be at higher risk of CHD. The important strengths of this clinical study included the prospective study design, the large sample size, the well-characterized population, a wide range of values of light at night, and adjustment for a number of individual and neighborhood-level covariates. The main limitations of this study were the measure of outdoor light at night, lack of data of sleep quality, and weakness of generalization in younger ages [13].

Grimaldi et al. [49] aimed to determine the impact of circadian misalignment on autonomic nervous system control of cardiovascular function. Sleep restriction with circadian misalignment significantly increased urinary norepinephrine levels and reduced nocturnal heart rate variability. Secondly, sleep restriction with circadian misalignment elicited greater increases in nocturnal heart rate when compared to sleep restriction alone. Third, these alterations in autonomic indices did not translate into an augmentation of arterial BP, suggesting that other compensatory mechanisms. These findings demonstrated a clear adverse impact of circadian misalignment on autonomic function in sleep-restricted healthy adults [49].

Vetter et al. [50] examined the association of rotating night shift work with CHD incidence, over 24 years of follow-up and found that ≥5 years of rotating night shift work was associated with a significantly increased risk of CHD. Among the main strengths of this study were the number of cases over 24 years of follow-up. However, its findings cannot be generalized in men. Furthermore, studying CHD-related biomarkers could be useful in understanding underlying mechanisms [50].

Huang et al. [51] aimed to prospectively assess the association between sleep regularity and risk of CVD. This clinical study evaluated sleep regularity and the CVD incidence. They found that individuals with the most irregular sleep duration or timing had >2-fold risk of developing CVD over a median follow-up of 4.9 years compared with individuals with the most regular sleep patterns. One of the mechanisms through which irregular sleep influenced cardiovascular risk was the circadian clock genes. Moreover, the above findings remained robust after considering multiple established CVD risk factors and conventional measures of sleep quantity and quality, suggesting that irregular sleep duration and timing may be novel and independent risk factors for CVD. The main strength of this clinical study was its prospective design, but a minus was the modest sample size. Further research is needed to determine the extent to which increased sleep variability could influence CVD via effects on circadian disruption versus exposing individuals to intermittent short sleep that may inadequately be compensated for by over-sleeping [51].

## 4. Conclusions

Several clinical studies have been performed in order to investigate the interrelationship between circadian disruption and sleep, metabolic, and cardiovascular disorders. Based on the cumulative evidence on diseases related to circadian rhythmicity, these relationships appear to be both bidirectional and disease dependent.

As it is well recognized, the circadian rhythm affects the function of different systems such as cardiovascular, metabolic, neurological, and immunological system [52]. These functions are controlled by cyclical peaks and troughs in the production and levels of different hormones and biological functions [53]. Circadian rhythms are an important component of human physiology and are linked to almost all diseases [54]. Many of these diseases have a 24-h rhythm in an incidence and a disease burden [55]. Circadian misalignment has an impact on some physiological markers, such as hormone levels, blood pressure, and immune markers [56,57].

Consequences of the circadian asynchrony are faced by the night-shift workers. The International Agency for Research on Cancer (IARC) has defined that night-shift work is probably carcinogenic to humans. However, the results in bibliography are controversial [58]. A meta-analyses of 31 prospective cohort studies supported that night-shift work exposure significantly increased the risk of breast cancer morbidity by 2.9% for total, 8.6% for the subgroup of more than 10 years night-shift work, and 5.3% for rotating night-shift work [59]. A different meta-analysis of 26 eligible studies found no increase in the long-term night-shift workers [60]. In addition, disruption in circadian processes influences many physiological functions, including female reproduction. In fact, an important association has been observed between infertility in young women and night shift work due to impaired immune system and inflammatory responses [61]. Environmental factors that disrupt physiological rhythms might also contribute to cardiovascular events, as well as increasing other risk factors typically associated with CVD [2,62,63].

Shift work and certain sleep disorders like insomnia, obstructive sleep apnea, and reduced sleep and other factors like jet lag and timing of food intake can cause a state of circadian disruption, which increases the risk of developing metabolic and cardiovascular diseases [64,65,66,67,68,69]. For instance, clock genes are expressed cell-autonomously that receive projections from the suprachiasmatic nucleus (SCN); thus, it is possible that oscillation of key transcripts important in the response to anorexigenic and orexigenic hormones may be subject to circadian control [17,70].

There is increasing evidence of detrimental effects on metabolic function and dietary choices, emphasizing the importance of bolstering circadian system function and addressing sleep disruption [71,72,73]. Nowadays, it is common to be exposed to less light during the day and more light at night because of artificial lighting, which may impair circadian system organization and disrupt sleep, resulting in widespread adverse effects on metabolic health [74,75,76]. As indicated by strong epidemiological and laboratory evidence, the circadian cycle disturbance not only causes sleep problems, but further disrupts the optimal rhythm of many physiological functions, thus increasing the risk of cardiometabolic and other related diseases [77,78,79]. Circadian misalignment increases inflammation and impairs glucose regulation, with potential relevance to CDVs and slows the recovery of them [12]. Therefore, it should be clear that, by addressing the disturbances that cause circadian misalignment, the risk of CVD can be reduced [4,78,80].

According to the literature research, the strengths of the currently available clinical studies that were included in this article highlight the carefully designed experimental protocols and the range of the examined metabolic parameters.

If we could summarize the main limitations of the existing clinical evidence, the cross-sectional nature of the eligible studies cannot support causality. Moreover, we could add the following ones: the small sample size and the short duration of the interventions. Finally, a common barrier was the existence of sex limitations because only few studies included male volunteers.

Although we performed a thorough search for the last 23 years (from 2000 to 2023), the vast majority of the existing studies in this topic has been performed just the last decade, since it constitutes a very novel scientific area. In this aspect, further research should continue to investigate the causal relationships and then determine the role of circadian disorder in many related diseases. The concept of circadian hygiene can be useful for implementing interventions and programs in different settings. Broader than the concept of sleep hygiene, circadian hygiene-based interventions and programs would have to take into account necessary changes in every aspect of social life [81,82]. Understanding the underlying mechanisms that regulate circadian rhythm may provide insights into circadian physiology and advance novel chronotherapy approaches and therapeutic targets for metabolic disorders [83,84,85].

As far as the development of prevention interventional strategies and policies against metabolic and cardiovascular diseases is concerned, there is little clinical humans’ evidence regarding the recommendation of circadian rhythms and sleep to prevent metabolic and cardiovascular disorders. In this aspect, melatonin, a neuroendocrine hormone with critical physiological roles in the circadian rhythm and sleep–wake cycle, may exert beneficial effects in the cardiovascular system mainly through its antioxidant activity [86]. Notably, a recent meta-analysis noted that melatonin supplementation could be effective in improving lipid parameters and may be utilized in the prevention of CVD, although the effect of this supplement on anthropometric indices was not significant and needs further investigation [87]. Overall, disturbed sleep and circadian rhythms may represent modifiable risk factors for the prevention and treatment of metabolic and cardiovascular diseases and for the promotion of healthy metabolism and cardiovascular system.

## Figures and Tables

**Table 1:** Clinical studies analyzing the relationship between circadian rhythms and metabolic diseases.

Study Type	Study Population	Basic Results	References
Clinical Trial	N = 26 adults	Circadian misalignment can have adverse effects on insulin action, insulin release, melatonin and high sensitive c-reactive protein (hsCRP).	Leproult et al. [30]
Clinical Trial	N = 14 adults	Glucose tolerance was 17% lower in the biological evening than in the biological morning on test day 1. Circadian misalignment itself increased postprandial glucose by 6%.	Morris et al. [31]
Clinical Trial	N = 9 adults	Circadian misalignment increased 24-h systolic blood pressure (SBP) and diastolic blood pressure (DBP) by 3.0 mmHg and 1.5 mmHg, respectively. Circadian misalignment decreased wake cardiac vagal modulation by 8–15%, as determined by heart rate variability analysis, and decreased 24-h urinary epinephrine excretion rate by 7%, without a significant effect on 24-h urinary norepinephrine excretion rate. Circadian misalignment increased 24-h serum interleukin-6, CRP, resistin, and TNF-α levels by 3–29%.	Morris et al. [32]
Randomized Controlled Trial	N = 9 adults	Circadian misalignment per se increased hs-CRP and blood pressure in shift workers.	Morris et al. [33]
Randomized Controlled Trial	N = 14 adults	Circadian phase and circadian misalignment affected glucose tolerance.	Qian et al. [34]
Randomized Clinical Trial	N = 14 adult (men)	Circadian misalignment also led to higher fasting free fatty acid (FFA) levels, fasting plasma glucose levels, and lower muscle insulin sensitivity and triglyceride levels.	Wefers et al. [35]
Randomized Controlled Trial	N = 80, adults (overweight and obese women)	Compared with the Dinner Meal group, the Lunch Meal group had greater mean reductions in weight, body mass index (BMI), homeostasis model assessment of insulin resistance (HOMA-IR) and fasting insulin after 12 wks.	Madjd et al. [36]
Randomized Controlled Trial	N = 32, adults (women)	Late eating resulted in a significantly lower pre-meal utilization of carbohydrates and decreased glucose tolerance.	Bandín et al. [37]
Randomized Controlled Trial	Ν = 13.026, adults	Patients who reported <6 h sleep per night had a 29% higher risk of major coronary events (MCE) compared with those with longer sleep. Patients who screened positive for obstructive sleep apnea had a 12% higher risk of MCE than those who did not screen positive. Overnight shift work (≥3-night shifts/week for ≥1 year) was associated with a 15% higher risk of MCE.	Barger et al. [38]
Randomized Controlled Trial	N = 12 adults	Postprandial glycemic excursion was higher during the night shift. The time to peak insulin and C-peptide and nadir glucagon suppression in response to meal ingestion was delayed during the night shift.	Sharma et al. [39]
Clinical Trial	Ν = 180 adults	Insomnia might trigger a nocturnal stress response within the nervous and endocrine system, expediting the progression of cardiovascular morbidity.	Jarrin et al. [40]

## Data Availability

Data is not publicly available due to privacy.

## References

[1] Man A., Xia N., Li H. (2020). Circadian Rhythm in Adipose Tissue: Novel Antioxidant Target for Metabolic and Cardiovascular Diseases. Antioxidants.

[2] Fishbein A.B., Knutson K.L., Zee P.C. (2021). Circadian disruption and human health. J. Clin. Investig..

[3] Jagannath A., Taylor L., Wakaf Z., Vasudevan S.R., Foster R.G. (2017). The genetics of circadian rhythms, sleep and health. Hum. Mol. Genet..

[4] Khan S., Malik B.H., Gupta D., Rutkofsky I. (2020). The Role of Circadian Misalignment due to Insomnia, Lack of Sleep, and Shift Work in Increasing the Risk of Cardiac Diseases: A Systematic Review. Cureus.

[5] Reid K.J. (2019). Assessment of Circadian Rhythms. Neurol. Clin..

[6] Dierickx P., Van Laake L.W., Geijsen N. (2018). Circadian clocks: From stem cells to tissue homeostasis and regeneration. EMBO Rep..

[7] Nakanishi-Minami T., Kishida K., Funahashi T., Shimomura I. (2012). Sleep-wake cycle irregularities in type 2 diabetics. Diabetol. Metab. Syndr..

[8] Depner C.M., Stothard E.R., Wright K.P. (2014). Metabolic consequences of sleep and circadian disorders. Curr. Diabetes Rep..

[9] Woller A., Gonze D. (2021). Circadian Misalignment and Metabolic Disorders: A Story of Twisted Clocks. Biology.

[10] Khosravipour M., Khanlari P., Khazaie S., Khosravipour H., Khazaie H. (2021). A systematic review and meta-analysis of the association between shift work and metabolic syndrome: The roles of sleep, gender, and type of shift work. Sleep Med. Rev..

[11] Crnko S., Du Pré B.C., Sluijter J.P.G., Van Laake L.W. (2019). Circadian rhythms and the molecular clock in cardiovascular biology and disease. Nat. Rev. Cardiol..

[12] Thosar S.S., Butler M.P., Shea S.A. (2018). Role of the circadian system in cardiovascular disease. J. Clin. Investig..

[13] Sun S., Cao W., Ge Y., Ran J., Sun F., Zeng Q., Guo M., Huang J., Lee R.S., Tian L. (2021). Outdoor light at night and risk of coronary heart disease among older adults: A prospective cohort study. Eur. Heart J..

[14] Klerman E.B., Brager A., Carskadon M.A., Depner C.M., Foster R., Goel N., Harrington M., Holloway P.M., Knauert M.P., LeBourgeois M.K. (2022). Keeping an eye on circadian time in clinical research and medicine. Clin. Transl. Med..

[15] McHill A.W., Wright K.P. (2017). Role of sleep and circadian disruption on energy expenditure and in metabolic predisposition to human obesity and metabolic disease. Obes. Rev. Off. J. Int. Assoc. Study Obes..

[16] Chellappa S.L., Vujovic N., Williams J.S., Scheer F. (2019). Impact of Circadian Disruption on Cardiovascular Function and Disease. Trends Endocrinol. Metab. TEM.

[17] Huang W., Ramsey K.M., Marcheva B., Bass J. (2011). Circadian rhythms, sleep, and metabolism. J. Clin. Investig..

[18] Dutheil F., Baker J.S., Mermillod M., De Cesare M., Vidal A., Moustafa F., Pereira B., Navel V. (2020). Shift work, and particularly permanent night shifts, promote dyslipidaemia: A systematic review and meta-analysis. Atherosclerosis.

[19] Kervezee L., Kosmadopoulos A., Boivin D.B. (2020). Metabolic and cardiovascular consequences of shift work: The role of circadian disruption and sleep disturbances. Eur. J. Neurosci..

[20] Vasey C., McBride J., Penta K. (2021). Circadian Rhythm Dysregulation and Restoration: The Role of Melatonin. Nutrients.

[21] Pavlova M. (2017). Circadian Rhythm Sleep-Wake Disorders. Contin. Lifelong Learn. Neurol..

[22] Zisapel N. (2001). Circadian rhythm sleep disorders: Pathophysiology and potential approaches to management. CNS Drugs.

[23] Gentry N.W., Ashbrook L.H., Fu Y.H., Ptáček L.J. (2021). Human circadian variations. J. Clin. Investig..

[24] Zee P.C., Abbott S.M. (2020). Circadian Rhythm Sleep-Wake Disorders. Continuum.

[25] Roenneberg T., Foster R.G., Klerman E.B. (2022). The circadian system, sleep, and the health/disease balance: A conceptual review. J. Sleep Res..

[26] Hittle B.M., Gillespie G.L. (2018). Identifying shift worker chronotype: Implications for health. Ind. Health.

[27] Stevens R.G., Brainard G.C., Blask D.E., Lockley S.W., Motta M.E. (2013). Adverse health effects of nighttime lighting: Comments on American Medical Association policy statement. Am. J. Prev. Med..

[28] Fatima N., Rana S. (2020). Metabolic implications of circadian disruption. Pflug. Arch. Eur. J. Physiol..

[29] Poggiogalle E., Jamshed H., Peterson C.M. (2018). Circadian regulation of glucose, lipid, and energy metabolism in humans. Metab. Clin. Exp..

[30] Leproult R., Holmbäck U., Van Cauter E. (2014). Circadian misalignment augments markers of insulin resistance and inflammation, independently of sleep loss. Diabetes.

[31] Morris C.J., Yang J.N., Garcia J.I., Myers S., Bozzi I., Wang W., Buxton O.M., Shea S.A., Scheer F.A. (2015). Endogenous circadian system and circadian misalignment impact glucose tolerance via separate mechanisms in humans. Proc. Natl. Acad. Sci. USA.

[32] Morris C.J., Purvis T.E., Mistretta J., Scheer F.A. (2016). Effects of the Internal Circadian System and Circadian Misalignment on Glucose Tolerance in Chronic Shift Workers. J. Clin. Endocrinol. Metab..

[33] Morris C.J., Purvis T.E., Mistretta J., Hu K., Scheer F. (2017). Circadian Misalignment Increases C-Reactive Protein and Blood Pressure in Chronic Shift Workers. J. Biol. Rhythm..

[34] Qian J., Dalla Man C., Morris C.J., Cobelli C., Scheer F. (2018). Differential effects of the circadian system and circadian misalignment on insulin sensitivity and insulin secretion in humans. Diabetes Obes. Metab..

[35] Wefers J., van Moorsel D., Hansen J., Connell N.J., Havekes B., Hoeks J., van Marken Lichtenbelt W.D., Duez H., Phielix E., Kalsbeek A. (2018). Circadian misalignment induces fatty acid metabolism gene profiles and compromises insulin sensitivity in human skeletal muscle. Proc. Natl. Acad. Sci. USA.

[36] Madjd A., Taylor M.A., Delavari A., Malekzadeh R., Macdonald I.A., Farshchi H.R. (2016). Beneficial effect of high energy intake at lunch rather than dinner on weight loss in healthy obese women in a weight-loss program: A randomized clinical trial. Am. J. Clin. Nutr..

[37] Bandín C., Scheer F.A., Luque A.J., Ávila-Gandía V., Zamora S., Madrid J.A., Gómez-Abellán P., Garaulet M. (2015). Meal timing affects glucose tolerance, substrate oxidation and circadian-related variables: A randomized, crossover trial. Int. J. Obes..

[38] Barger L.K., Rajaratnam S.M.W., Cannon C.P., Lukas M.A., Im K., Goodrich E.L., Czeisler C.A., O’Donoghue M.L. (2017). Short Sleep Duration, Obstructive Sleep Apnea, Shiftwork, and the Risk of Adverse Cardiovascular Events in Patients After an Acute Coronary Syndrome. J. Am. Heart Assoc..

[39] Sharma A., Laurenti M.C., Dalla Man C., Varghese R.T., Cobelli C., Rizza R.A., Matveyenko A., Vella A. (2017). Glucose metabolism during rotational shift-work in healthcare workers. Diabetologia.

[40] Jarrin D.C., Ivers H., Lamy M., Chen I.Y., Harvey A.G., Morin C.M. (2018). Cardiovascular autonomic dysfunction in insomnia patients with objective short sleep duration. J. Sleep Res..

[41] Wong P.M., Hasler B.P., Kamarck T.W., Muldoon M.F., Manuck S.B. (2015). Social Jetlag, Chronotype, and Cardiometabolic Risk. J. Clin. Endocrinol. Metab..

[42] Ritonja J., Tranmer J., Aronson K.J. (2019). The relationship between night work, chronotype, and cardiometabolic risk factors in female hospital employees. Chronobiol. Int..

[43] Hulsegge G., Picavet H.S.J., van der Beek A.J., Verschuren W.M.M., Twisk J.W., Proper K.I. (2019). Shift work, chronotype and the risk of cardiometabolic risk factors. Eur. J. Public Health.

[44] Yu J.H., Yun C.H., Ahn J.H., Suh S., Cho H.J., Lee S.K., Yoo H.J., Seo J.A., Kim S.G., Choi K.M. (2015). Evening chronotype is associated with metabolic disorders and body composition in middle-aged adults. J. Clin. Endocrinol. Metab..

[45] Zhang R., Cai X., Lin C., Yang W., Lv F., Wu J., Ji L. (2022). The association between metabolic parameters and evening chronotype and social jetlag in non-shift workers: A meta-analysis. Front. Endocrinol..

[46] Iwasaki M., Hirose T., Mita T., Sato F., Ito C., Yamamoto R., Someya Y., Yoshihara T., Tamura Y., Kanazawa A. (2013). Morningness-eveningness questionnaire score correlates with glycated hemoglobin in middle-aged male workers with type 2 diabetes mellitus. J. Diabetes Investig..

[47] Koopman A.D.M., Rauh S.P., van ’t Riet E., Groeneveld L., van der Heijden A.A., Elders P.J., Dekker J.M., Nijpels G., Beulens J.W., Rutters F. (2017). The Association between Social Jetlag, the Metabolic Syndrome, and Type 2 Diabetes Mellitus in the General Population: The New Hoorn Study. J. Biol. Rhythm..

[48] Estarlich M., Tolsa C., Trapero I., Buigues C. (2022). Circadian Variations and Associated Factors in Patients with Ischaemic Heart Disease. Int. J. Environ. Res. Public Health.

[49] Grimaldi D., Carter J.R., Van Cauter E., Leproult R. (2016). Adverse Impact of Sleep Restriction and Circadian Misalignment on Autonomic Function in Healthy Young Adults. Hypertens.

[50] Vetter C., Devore E.E., Wegrzyn L.R., Massa J., Speizer F.E., Kawachi I., Rosner B., Stampfer M.J., Schernhammer E.S. (2016). Association between Rotating Night Shift Work and Risk of Coronary Heart Disease Among Women. Jama.

[51] Huang T., Mariani S., Redline S. (2020). Sleep Irregularity and Risk of Cardiovascular Events: The Multi-Ethnic Study of Atherosclerosis. J. Am. Coll. Cardiol..

[52] Mattis J., Sehgal A. (2016). Circadian Rhythms, Sleep, and Disorders of Aging. Trends Endocrinol. Metab. TEM.

[53] Roenneberg T., Merrow M. (2016). The Circadian Clock and Human Health. Curr. Biol. CB.

[54] Man A.W.C., Li H., Xia N. (2021). Circadian Rhythm: Potential Therapeutic Target for Atherosclerosis and Thrombosis. Int. J. Mol. Sci..

[55] Meléndez-Fernández O.H., Walton J.C., DeVries A.C., Nelson R.J. (2021). Clocks, Rhythms, Sex, and Hearts: How Disrupted Circadian Rhythms, Time-of-Day, and Sex Influence Cardiovascular Health. Biomolecules.

[56] Hemmer A., Mareschal J., Dibner C., Pralong J.A., Dorribo V., Perrig S., Genton L., Pichard C., Collet T.H. (2021). The Effects of Shift Work on Cardio-Metabolic Diseases and Eating Patterns. Nutrients.

[57] Yamanaka Y. (2020). Basic concepts and unique features of human circadian rhythms: Implications for human health. Nutr. Rev..

[58] Dun A., Zhao X., Jin X., Wei T., Gao X., Wang Y., Hou H. (2020). Association between Night-Shift Work and Cancer Risk: Updated Systematic Review and Meta-Analysis. Front. Oncol..

[59] Wei F., Chen W., Lin X. (2022). Night-shift work, breast cancer incidence, and all-cause mortality: An updated meta-analysis of prospective cohort studies. Sleep Breath. Schlaf Atm..

[60] Manouchehri E., Taghipour A., Ghavami V., Ebadi A., Homaei F., Latifnejad Roudsari R. (2021). Night-shift work duration and breast cancer risk: An updated systematic review and meta-analysis. BMC Women’s Health.

[61] Fernandez R.C., Moore V.M., Marino J.L., Whitrow M.J., Davies M.J. (2020). Night Shift Among Women: Is It Associated With Difficulty Conceiving a First Birth?. Front. Public Health.

[62] Peñaloza-Martínez E., Moreno G., Aroca-Crevillén A., Huertas S., Vicent L., Rosillo N., Hidalgo A., Bueno H. (2022). Circadian rhythms in thrombosis and atherothrombotic events. Front. Biosci. Landmark Ed..

[63] Stubblefield J.J., Lechleiter J.D. (2019). Time to Target Stroke: Examining the Circadian System in Stroke. Yale J. Biol. Med..

[64] Baron K.G., Reid K.J. (2014). Circadian misalignment and health. Int. Rev. Psychiatry.

[65] Broussard J.L., Van Cauter E. (2016). Disturbances of sleep and circadian rhythms: Novel risk factors for obesity. Curr. Opin. Endocrinol. Diabetes Obes..

[66] Chaput J.P., McHill A.W., Cox R.C., Broussard J.L., Dutil C., da Costa B.G.G., Sampasa-Kanyinga H., Wright K.P. (2023). The role of insufficient sleep and circadian misalignment in obesity. Nat. Rev. Endocrinol..

[67] Abbott S.M., Malkani R.G., Zee P.C. (2020). Circadian disruption and human health: A bidirectional relationship. Eur. J. Neurosci..

[68] Meyer N., Harvey A.G., Lockley S.W., Dijk D.J. (2022). Circadian rhythms and disorders of the timing of sleep. Lancet.

[69] Belloir J., Makarem N., Shechter A. (2022). Sleep and Circadian Disturbance in Cardiovascular Risk. Curr. Cardiol..

[70] Boivin D.B., Boudreau P., Kosmadopoulos A. (2022). Disturbance of the Circadian System in Shift Work and Its Health Impact. J. Biol. Rhythm..

[71] Vetter C. (2020). Circadian disruption: What do we actually mean?. Eur. J. Neurosci..

[72] Chasens E.R., Imes C.C., Kariuki J.K., Luyster F.S., Morris J.L., DiNardo M.M., Godzik C.M., Jeon B., Yang K. (2021). Sleep and Metabolic Syndrome. Nurs. Clin. North Am..

[73] Steele T.A., St Louis E.K., Videnovic A., Auger R.R. (2021). Circadian Rhythm Sleep-Wake Disorders: A Contemporary Review of Neurobiology, Treatment, and Dysregulation in Neurodegenerative Disease. Neurotherapeutics.

[74] Potter G.D., Skene D.J., Arendt J., Cade J.E., Grant P.J., Hardie L.J. (2016). Circadian Rhythm and Sleep Disruption: Causes, Metabolic Consequences, and Countermeasures. Endocr. Rev..

[75] Sooriyaarachchi P., Jayawardena R., Pavey T., King N.A. (2022). Shift work and the risk for metabolic syndrome among healthcare workers: A systematic review and meta-analysis. Obes. Rev. Off. J. Int. Assoc. Study Obes..

[76] Cable J., Schernhammer E., Hanlon E.C., Vetter C., Cedernaes J., Makarem N., Dashti H.S., Shechter A., Depner C., Ingiosi A. (2021). Sleep and circadian rhythms: Pillars of health-a Keystone Symposia report. Ann. N. Y. Acad. Sci..

[77] Korsiak J., Tranmer J., Day A., Aronson K.J. (2018). Sleep duration as a mediator between an alternating day and night shift work schedule and metabolic syndrome among female hospital employees. Occup. Environ. Med..

[78] Zimmet P., Alberti K., Stern N., Bilu C., El-Osta A., Einat H., Kronfeld-Schor N. (2019). The Circadian Syndrome: Is the Metabolic Syndrome and much more!. J. Intern. Med..

[79] Peng F., Li X., Xiao F., Zhao R., Sun Z. (2022). Circadian clock, diurnal glucose metabolic rhythm, and dawn phenomenon. Trends Neurosci..

[80] Parameswaran G., Ray D.W. (2022). Sleep, circadian rhythms, and type 2 diabetes mellitus. Clin. Endocrinol..

[81] Moreno C.R.C., Raad R., Gusmão W.D.P., Luz C.S., Silva V.M., Prestes R.M., Saraiva S.P., Lemos L.C., Vasconcelos S.P., Nehme P. (2022). Are We Ready to Implement Circadian Hygiene Interventions and Programs?. Int. J. Environ. Res. Public Health.

[82] Hower I.M., Harper S.A., Buford T.W. (2018). Circadian Rhythms, Exercise, and Cardiovascular Health. J. Circadian Rhythm..

[83] Logan R.W., McClung C.A. (2019). Rhythms of life: Circadian disruption and brain disorders across the lifespan. Nat. Reviews. Neurosci..

[84] Ruan W., Yuan X., Eltzschig H.K. (2021). Circadian rhythm as a therapeutic target. Nat. Rev. Drug Discov..

[85] Vandenberghe A., Lefranc M., Furlan A. (2022). An Overview of the Circadian Clock in the Frame of Chronotherapy: From Bench to Bedside. Pharmaceutics.

[86] Tobeiha M., Jafari A., Fadaei S., Ali Mirazimi S.M., Dashti F., Amiri A., Khan H., Asemi Z., Reiter R.J., Hamblin M.R. (2022). Evidence for the Benefits of Melatonin in Cardiovascular Disease. Front. Cardiovasc. Med..

[87] Loloei S., Sepidarkish M., Heydarian A., Tahvilian N., Khazdouz M., Heshmati J., Pouraram H. (2019). The effect of melatonin supplementation on lipid profile and anthropometric indices: A systematic review and meta-analysis of clinical trials. Diabetes Metab. Syndr..

